# Breastfeeding and the risk of breast cancer in *BRCA1 *and *BRCA2 *mutation carriers

**DOI:** 10.1186/bcr3138

**Published:** 2012-03-09

**Authors:** Joanne Kotsopoulos, Jan Lubinski, Leonardo Salmena, Henry T Lynch, Charmaine Kim-Sing, William D Foulkes, Parviz Ghadirian, Susan L Neuhausen, Rochelle Demsky, Nadine Tung, Peter Ainsworth, Leigha Senter, Andrea Eisen, Charis Eng, Christian Singer, Ophira Ginsburg, Joanne Blum, Tomasz Huzarski, Aletta Poll, Ping Sun, Steven A Narod

**Affiliations:** 1 Familial Breast Cancer Unit, Women's College Research Institute, 790 Bay Street, Toronto, ON M5G 1N8, Canada; 2 Hereditary Cancer Center, Pomeranian Medical University, Ul Polabska 4, 70-115 Szczecin, Poland; 3 Department of Preventive Medicine and Public Health, Creighton University School of Medicine, 2500 California Plaza, Omaha, NE 68178 USA; 4 BC Cancer Agency, 600 10th Ave. W, Vancouver, BC V5Z 4E6, Canada; 5 Programs in Cancer Genetics, Department of Oncology and Human Genetics, McGill University, 3755 Côte Ste-Catherine Montréal, QC H3T 1E2, Canada; 6 Epidemiology Research Unit, Research Center of the University of Montreal Hospital Centre (CRCHUM), 3850, rue Saint-Urbain Pavillon Masson, Montreal, QC H2W 1T7, Canada; 7 Department of Population Sciences, Beckman Research Institute of City of Hope, 1500 East Duarte Rd., Duarte, CA 91010, USA; 8 Division of Gynecologic Oncology, Department of Obstetrics and Gynecology, University of Toronto, 92 College Street, Toronto, ON M5G 1L4, Canada; 9 Beth Israel Deaconess Hospital, 330 Brookline, Boston, MA 02215, USA; 10 London Regional Cancer Program, 800 Commissioners Rd. E., London, ON N6A 5W9, Canada; 11 Division of Human Genetics, The Ohio State University Medical Center, Comprehensive Cancer Center, 2001 Polaris Parkway, Columbus, OH 43240, USA; 12 Toronto-Sunnybrook Regional Cancer Center, 2075 Bayview Ave., Toronto, ON M4N 3M5, Canada; 13 Genomic Medicine Institute and Center for Personalized Genetic Healthcare, Cleveland Clinic, 9500 Euclid Ave., Cleveland, OH 44195, USA; 14 Department of Obstetrics and Gynecology and Comprehensive Cancer Center, Medical University of Vienna, Spitalgasse 23, 1090 Wien, Austria; 15 Department of Medicine, University of Toronto, 1 Kings College Circle, Toronto, ON M5S 1A8, Canada; 16 Baylor University Medical Center, Hereditary Cancer Risk Program, 3535 Worth Street, Dallas, TX 75246, USA

## Abstract

**Introduction:**

Breastfeeding has been inversely related to breast cancer risk in the general population. Clarifying the role of breastfeeding among women with a *BRCA1 *or *BRCA2 *mutation may be helpful for risk assessment and for recommendations regarding prevention. We present an updated analysis of breastfeeding and risk of breast cancer using a large matched sample of *BRCA *mutation carriers.

**Methods:**

We conducted a case-control study of 1,665 pairs of women with a deleterious mutation in either *BRCA1 *(*n *= 1,243 pairs) or *BRCA2 *(*n *= 422 pairs). Breast cancer cases and unaffected controls were matched on year of birth, mutation status, country of residence and parity. Information about reproductive factors, including breastfeeding for each live birth, was collected from a routinely administered questionnaire. Conditional logistic regression was used to estimate the association between ever having breastfed, as well as total duration of breastfeeding, and the risk of breast cancer.

**Results:**

Among *BRCA1 *mutation carriers, breastfeeding for at least one year was associated with a 32% reduction in risk (OR = 0.68; 95% CI 0.52 to 0.91; *P *= 0.008); breastfeeding for two or more years conferred a greater reduction in risk (OR = 0.51; 95% CI 0.35 to 0.74). Among *BRCA2 *mutation carriers, there was no significant association between breastfeeding for at least one year and breast cancer risk (OR = 0.83; 95% CI 0.53 to 1.31; *P *= 0.43).

**Conclusions:**

These data extend our previous findings that breastfeeding protects against *BRCA1*-, but not *BRCA2*-associated breast cancer. *BRCA *mutation carriers should be advised of the benefit of breastfeeding in terms of reducing breast cancer risk.

## Introduction

In the general population, reproductive factors, including late age at menarche, parity and breastfeeding, have been shown to protect against the development of breast cancer [1-3]. Various proposed mechanisms include reducing lifetime exposure to ovarian hormones, reducing the cumulative number of ovulatory cycles and differentiation of the breast lobules [4,5]. We and others have evaluated the impact of reproductive factors in the etiology of *BRCA-*associated breast cancer, although the results are conflicting and vary by *BRCA1 *or *BRCA2 *mutation [6-8]. With respect to breastfeeding and breast cancer risk in *BRCA1 *mutation carriers, two previous studies reported no relationship [9,10] and three studies reported a protective effect [11,12]. One case-control study of *BRCA2 *mutation carriers reported no association between breastfeeding and breast cancer risk [8].

We have previously reported in a study of 965 matched pairs that the total duration of breastfeeding was associated with a significant reduction in breast cancer risk among *BRCA1*, but not among *BRCA2*, mutation carriers [11]. The association was particularly strong for breastfeeding for more than one year (OR = 0.55; 95% CI 0.38 to 0.80). No association was seen for *BRCA2 *mutation carriers (OR = 0.95; 95% CI 0.59 to 1.59). Here we update the analysis of total duration of breastfeeding and risk of breast cancer using a larger sample of *BRCA *mutation carriers with the addition of 700 matched pairs. Because childbirth and breastfeeding are strongly correlated, we restricted the present analysis to parous women and we matched on parity to eliminate any potential confounding effect of parity.

## Methods

### Study population

Eligible study subjects were identified at one of 62 participating centers in seven countries. These women were participants in ongoing research protocols at the host institutions. These women sought testing for *BRCA1 *and *BRCA2 *mutations because of a personal and/or family history of breast and/or ovarian cancer. All study subjects, (with the exception of some participants from the research study of SLN), received genetic counselling. The institutional review boards of the host institutions approved the study. All subjects provided written informed consent. In most cases, testing was initially offered to women who had been previously diagnosed with breast or ovarian cancer. When a *BRCA1 *or *BRCA2 *mutation was identified in a proband or her relative, genetic testing was offered to other at-risk individuals in the family. Mutation detection was performed using a range of techniques, but all nucleotide sequences were confirmed by direct sequencing of DNA. A woman was eligible for the current study when the molecular analysis established that she was a carrier of a deleterious mutation in the *BRCA1 *or *BRCA2 *gene.

### Data collection

All study subjects completed a baseline questionnaire at the individual center at the time of a clinic appointment or at their home at a later date. The questionnaire requested information on family and personal history of cancer, reproductive and medical histories, including preventive oophorectomy and mastectomy. Detailed information regarding ages at menarche and menopause, cause of menopause, pregnancy, breastfeeding history, and hormone use was also queried. For each live birth, women were asked to recall how long they breastfed each child and to indicate the number of months of breastfeeding. We estimated the total duration of breastfeeding for each woman by summing the months of breastfeeding for each live birth.

### Case and control subjects

Information on cancer status was available for a total of 12,116 women who carried a *BRCA1 *or *BRCA2 *mutation. Case subjects were women with a diagnosis of invasive breast cancer. Control subjects were women who never had breast cancer and who were also carriers of a mutation in *BRCA1 *or *BRCA2*. Potential subjects were excluded if they were nulliparous (*n *= 2,626) or if information on parity (including year of last birth) was missing (*n *= 320), if they had been diagnosed with ovarian or other cancer (*n *= 2,325), if information on breastfeeding was missing (*n *= 721), if *BRCA *mutation status was missing (*n *= 191) or if other pertinent information was missing (*n *= 54). After exclusions, there was a total of 5,879 eligible women, including 2,784 women with breast cancer (potential case subjects) and 3,095 women without breast cancer (potential control subjects).

A single control subject was selected for each case subject, matched according to mutation in the same gene (*BRCA1 *or *BRCA2*), year of birth (within one year), country of residence (Poland, USA, Canada, Israel, Austria, Italy, United Kingdom), and parity (number of births). We only included live births one or more years prior to the breast cancer diagnosis of the case for both the cases and controls. A control was eligible to be matched to a given case if the date of interview or date of prophylactic mastectomy in the matched control occurred at or after the year of breast cancer diagnosis of the case and if the last live birth of the control was at least one calendar year prior to the year of breast cancer diagnosis of the case. In total, 1,665 matched sets were identified. An appropriate match could not be located for 1,119 of the eligible cases because the date of birth of these cases (mean year of birth 1947.99; range 1901 to 1981) was substantially earlier compared to the pool of remaining controls (mean year of birth 1966.57; range 1903 to 1989).

### Statistical analysis

A matched case-control analysis was performed to evaluate the association between breastfeeding and the risk of breast cancer. We censored breastfeeding one year prior to the diagnosis of the matched case. The distributions of continuous and categorical variables between cases and controls were compared using the Student's *t*-test and chi-square test, respectively. Conditional logistic regression was used to estimate the univariate odds ratios (OR) and 95% confidence intervals (CI) for breast cancer associated with breastfeeding (ever/never) and total duration of breastfeeding (months). A multivariate analysis was carried out to control for potential confounders. All analyses were performed using the SAS statistical package, version 9.1.3 (SAS Institute, Cary, NC, USA). All *P-*values were based on two-sided tests and were considered statistically significant if *P *≤ 0.05.

## Results

Case and control subjects were similar with respect to year of birth, oral contraceptive use, age at last birth, smoking status and body mass index (BMI) at age 30 (Table 1). Cases with a *BRCA1 *mutation had a significantly earlier age at menarche than controls (13.09 years vs. 13.27 years; *P *= 0.003) and were less likely to have consumed alcohol (61% vs. 67%; *P *= 0.002). Although not significant, age at first birth (24.8 vs. 25.1; *P *= 0.08) and age at last birth (29.0 vs. 29.3; *P *= 0.06) were, on average, earlier in *BRCA1 *mutation carriers compared with controls. Hormone replacement therapy (HRT) use was significantly lower in both *BRCA1 *and *BRCA2 *cases versus controls (5% vs. 12%; *P *< 0.0001 and 10.7% vs. 16.5%; *P *= 0.02, respectively). Age at menarche, age at first birth, age at last birth, mean duration of breastfeeding and alcohol consumption did not differ between cases and controls with a *BRCA2 *mutation.

**Table 1:** Baseline characteristics of breast cancer cases and controls with *BRCA1 *and *BRCA2 *mutations.

Characteristic	*BRCA1*			*BRCA2*		
	Controls (n = 1,243)	Cases (n = 1,243)	*P*	Controls (n = 422)	Cases (n = 422)	*P*
Year of birth, mean (range)	1957.5 (1920-1980)	1957.3 (1920-1980)	0.71	1954.4 (1924-1978)	1955.3 (1924-1978)	0.82
Age at diagnosis, mean (range)	n/a^1^	0.71	n/a^1^	n/a^1^	42.7 (24-67)	n/a^1^
Country of residence, n (%)						
Poland	601 (48%)	matched		0 (0%)	matched	
USA	323 (26%)			187 (44%)		
Canada	265 (21%)			218 (52%)		
Israel	29 (2%)			13 (3%)		
Austria	18 (1.5%)			3 (0.7%)		
Italy	5 (0.4%)			0 (0%)		
United Kingdom	2 (0.2%)			2 (0.5%)		
Age at menarche, mean (range)	13.27(8-28)	13.09 (9-21)	0.003	12.64 (8-28)	12.15 (9-17)	0.88
Age at first birth, mean (range)	25.1 (15-44)	24.8 (15-42)	0.08	26.6 (17-44)	26.3 (16-42)	0.37
Age at last birth, mean (range)	29.3 (16-45)	29.0 (17-44)	0.06	30.5 (20-45)	30.2 (18-43)	0.96
Parity^2,4^, n (%)						
1	235 (19%)			65 (15%)		
2	663 (53%)			233 (55%)		
3	280 (23%)			100 (24%)		
≥ 4	65 (5%)			24 (6%)		
Mean (range)	2.16 (1-6)	matched		2.20 (1-6)	matched	
Breastfeeding, mean months^3,4 ^(range)	10.4 (0-92)	8.8 (0-84)	0.0009	9.7 (0-94)	10.2 (0-86)	0.56
Oral contraceptive use^4^, ever, n (%)	644 (52%)	657 (53%)	0.95	348 (83.5%)	336 (80.2%)	0.22
Smoking status, ever, n (%)	549 (45%)	574 (46%)	0.74	185 (44.4%)	170 (40.9%)	0.31
Alcohol use, ever, n (%)	832 (67%)	760 (61%)	0.002	305 (73.3%)	284 (68.8%)	0.13
HRT use^4^, n (%)	148 (12%)	67 (5%)	< 0.0001	69 (16.5%)	45 (10.7%)	0.02
BMI at age 30, mean (range)	22.5 (13-47)	22.6 (9-62)	0.24	22.8 (15-54)	23.0 (12-65)	0.48

^1^n/a, not applicable. ^2^Parity includes live births only. ^3^Breastfeeding is the total months of breastfeeding for each pregnancy combined. ^4^Parity, breastfeeding, oral contraceptive use and HRT use were censored one year prior to the diagnosis of the matched case for both cases and controls.

The mean duration of breastfeeding was shorter among the cases than the controls with a *BRCA1 *mutation (8.8 months vs. 10.4 months; *P *= 0.0009). There was a significant reduction in breast cancer risk with breastfeeding among women with a *BRCA1 *but not a *BRCA2 *mutation (Table 2). On average, *BRCA1 *cases breastfed for 1.6 fewer months than controls (8.8 vs. 10.4 months *P *= 0.0009). There was only a 0.5-month difference between cases and controls with a *BRCA2 *mutation (10.2 vs. 9.7 months *P *= 0.56). Among *BRCA1 *mutation carriers, breastfeeding for at least one year was associated with a 32% reduction in risk (OR = 0.68; 95% CI 0.52 to 0.91; *P *= 0.008); breastfeeding for two or more years conferred an even stronger reduction in risk (OR = 0.51; 95% CI 0.35 to 0.74; *P *= 0.0003). Each year of breastfeeding conferred a 19% reduction in risk. The effect of breastfeeding was present for women diagnosed at all ages. The reduction in risk associated with breastfeeding for one or more years compared with never breastfeeding was 44% for women with an age at breast cancer diagnosis of ≤ 39 (OR = 0.56; 95% CI 0.33 to 0.96), 54% for those with an age at diagnosis of 40 to 49 (OR = 0.46; 95% CI 0.26 to .81) and 69% for those with an age at diagnosis of ≥ 50 (OR = 0.31; 95% CI 0.07 to 1.30).

**Table 2:** Relationship between duration of breastfeeding and risk of breast cancer among *BRCA1 *and *BRCA2 *mutation carriers.

Breastfeeding duration	N (cases/controls)	Univariate OR (95% CI)	*P*	**Multivariate**^**1**^ OR (95% CI)	*P*
All subjects	1,665/1,665				
Never	389/327	1.00 (reference)		1.00 (reference)	
Ever	1,276/1,338	0.79 (0.67 to 0.94)	0.007	0.81 (0.68 to 0.97)	0.02
> 0 to ≤ 12 months	846/845	0.83 (0.69 to 0.99)	0.04	0.84 (0.70 to 1.02)	0.08
> 12 to ≤ 24 months	285/318	0.72 (0.58 to 0.90)	0.005	0.73 (0.58 to 0.93)	0.01
> 24 months	145/175	0.65 (0.49 to 0.86)	0.003	0.66 (0.49 to 0.89)	0.007
Per year		0.90 (0.84 to 0.97)	0.008	0.87 (0.79 to 0.95)	0.002
*BRCA1*	1,243/1,343				
Never	267/213	1.00 (reference)		1.00 (reference)	
Ever	976/1,030	0.74 (0.61 to 0.92)	0.005	0.76 (0.61 to 0.95)	0.02
> 0 to ≤ 12 months	669/658	0.79 (0.64 to 1.00)	0.03	0.81 (0.64 to 1.01)	0.06
> 12 to ≤ 24 months	212/236	0.68 (0.52 to 0.88)	0.004	0.68 (0.52 to 0.91)	0.008
> 24 months	94/136	0.50 (0.35 to 0.70)	< 0.0001	0.51 (0.35 to 0.74)	0.0003
Per year		0.84 (0.77 to 0.93)	0.0003	0.81 (0.73 to 0.91)	0.002
*BRCA2*	422/422				
Never	122/114	1.00 (reference)		1.00 (reference)	
Ever	300/308	0.91 (0.67 to 1.23)	0.53	0.90 (0.64 to 1.25)	0.51
> 0 to ≤ 12 months	177/187	0.89 (0.64 to 1.23)	0.47	0.89 (0.63 to 1.25)	0.49
> 12 to ≤ 24 months	72/82	0.84 (0.54 to 1.28)	0.40	0.83 (0.53 to 1.31)	0.43
> 24 months	51/39	1.21 (0.72 to 2.03)	0.48	1.20 (0.68 to 2.11)	0.53
Per year		1.05 (0.91 to 1.21)	0.51	1.02 (0.85 to 1.21)	0.87

^1^Estimate adjusted for age at menarche (continuous), age at first birth (continuous), age at first birth (continuous), alcohol consumption (ever/never) and HRT use (ever/never).

Among *BRCA2 *mutation carriers, there was no significant association between breastfeeding for at least one year and breast cancer risk (OR = 0.83; 95% CI 0.53 to 1.31; *P *= 0.43). There was no relationship following stratification by age at diagnosis. The risk estimates associated with breastfeeding for one or more years compared with never breastfeeding by age at diagnoses ≤ 39, 40 to 49 and ≥ 50 were 0.98 (95% CI 0.37 to 2.62), 1.41 (95% CI 0.59 to 3.43) and 0.91 (95% CI 0.24 to 3.43), respectively.

## Discussion

The goal of the current study was to evaluate the relationship between breastfeeding and breast cancer risk among women with a *BRCA1 *or *BRCA2 *mutation. Because of the strong relationship between parity and breastfeeding, we limited our analysis to parous women and we matched cases and controls on parity. Here we report that ever having breastfed and the total duration of breastfeeding conferred substantial reductions in breast cancer risk among *BRCA1*, but not *BRCA2 *mutation carriers. Breastfeeding for one or more years conferred a significant 32% reduction in risk in *BRCA1 *mutation carriers. These findings are in agreement with our earlier publication, which was based on a subset of these women [11]. In the present study, we expand our study sample from 995 to 1,665 matched pairs and we have matched on parity. The current study provides the most up-to-date evaluation of this association and should be helpful for genetic counsellors. The current study pertains to women who choose not to have a bilateral mastectomy; among women who have had preventive surgery, the risk of breast cancer is sufficiently low that they are not impacted adversely by the inability to breastfeed.

Other groups that have evaluated the relationship between breastfeeding and breast cancer risk among *BRCA *mutation carriers reported no relationship between breastfeeding and risk [8-10], although these studies were limited by small sample sizes and the merging of women who were carriers of a *BRCA1 *or *BRCA2 *mutation. These findings extend our previous report that breastfeeding for more than one year reduces risk among *BRCA1*, but not among *BRCA2 *mutation carriers [11].

In a collaborative re-analysis of data from 47 independent epidemiologic studies conducted among women in the general population, the authors reported a 4.3% reduction in breast cancer risk for every 12 months of breastfeeding (95% CI 2.9 to 5.8; *P *< 0.0001) [2]. The effect of breastfeeding among *BRCA1 *mutation carriers reported here is much greater than that reported in the general population (19% vs. 4.3% reduction in risk per year of breastfeeding). In *BRCA *mutation carriers specifically, it does not appear that parity *per se *influences the risk of breast cancer independently of breastfeeding. The majority of *BRCA1*-related tumors are basal-like breast cancers characterized by ER, PR and HER2 negativity while *BRCA2*-tumors resemble sporadic cases [13]. Other groups have shown that parity is a risk factor, while breastfeeding protects against the development of basal-like or ER-negative/PR-negative breast cancers in the general population [14-16]. Likewise, differences in the pathologic features of *BRCA1 *and *BRCA2 *tumors may also reflect etiologically distinct tumors and possibly differences in risk factor associations.

Several mechanisms have been proposed to explain the biologic basis for the inverse relationship between breastfeeding and breast cancer risk (reviewed in [17]). These include: 1) hormonal changes, particularly a reduction in endogenous estrogen and progesterone levels and/or increased prolactin levels; 2) excretion of estrogens and carcinogens from the breast ducts; 3) breast tissue differentiation; and 4) a delay in the establishment of regular ovulation and, subsequently, the cumulative number of ovulatory cycles [17]. We have recently shown that the number of lifetime ovulatory cycles does not influence breast cancer risk in women with *BRCA1 *or *BRCA2 *mutations (JK, JL, HTL, C K-S, SN, RD, WDF, PG, NT, PA, LS, Beth Karlan, AE, CE, Jeffrey Weitzel, DMG, JB, Dana Zakalik, CS, Taya Fallen, OG, TH, PS, SAN, Oophorectomy after Menopause and the Risk of Breast Cancer in *BRCA1 *and *BRCA2 *Mutation Carriers, submitted). Our data presented in this manuscript support the first three hypotheses and are reinforced by the long lasting effect of breastfeeding among *BRCA1 *mutation carriers. Breastfeeding for at least one year significantly reduced the risk of breast cancer diagnosed under the age of 50. Although the association did not achieve statistical significance (likely due to small numbers), this protective effect was also apparent for those diagnosed ≥ 50 and was indicative of a long-term reduction in risk with breastfeeding.

Experimental evidence supports an important role of the BRCA1 protein in mammary cell differentiation and proliferation. Rajan and colleagues have shown that *BRCA1 *and *BRCA2 *mRNA expression is high in proliferating cells in mice during periods of rapid proliferation and differentiation, such as embryogenesis, puberty and pregnancy, but is low during lactation [18-20]. In addition, the BRCA proteins interact with STAT5a, a mammary gland transcription factor that is stimulated by prolactin at the end of pregnancy, and which is involved in the growth and terminal differentiation of breast epithelial cells [21]. Thus, among women with a mutation and one functional allele, one would expect that breast cell differentiation from breastfeeding might be compromised.

*In vivo *studies have shown that a conditional mutation in *Brca1 *in mouse mammary epithelial cells results in defective development of mammary tissue during pregnancy and lactation [22,23]. Further, Russo *et al. *have reported that the developmental pattern of breast tissue from parous women with a family history of breast cancer or *BRCA1 *mutation was comparable to that from nulliparous women with no family history (that is, did not differentiate with parity) [24]. Collectively, the evidence supports an important role of the BRCA proteins in breast tissue differentiation during pregnancy and/or lactation.

Based on our findings to date, it appears that cumulative sex hormone exposure, particularly estrogen, is likely involved in the development of breast cancer in *BRCA1 *mutation carriers. Late age at menarche, breastfeeding and bilateral salpingo-oophorectomy are protective; while the role of parity is less clear. Recent studies have identified an important role for progesterone in regulating the number of stem cells in normal human and mouse mammary glands [25,26]. The terminal differentiation of mammary stem cells during the process of breastfeeding may be protective since this process can deplete the pool of potentially deleterious stem cells or by reducing the number of stem cells at risk of mutation. If confirmed, agents that can mimic the terminal differentiation process, or otherwise reduce the size of the stem cell population, may be effective in reducing breast cancer risk. Notably, it has been reported that heterozygosity of *BRCA1 *may result in abnormal self-renewal of breast cancer stem cells [27]. Specifically, failure of terminal differentiation after pregnancy in *BRCA1 *mutation carriers resulted in an increased risk of breast cancer [28].

Strengths of the current study include the large number of known *BRCA *mutation carriers and the ability to conduct a matched analysis to ensure that the case and control subjects were similar. The sample size range in four prior studies was 94 to 1,601 *BRCA *carriers [8-11]. To determine the effect of breastfeeding on cancer risk independent of the effects of parity, we limited our analysis to parous women and matched for parity. We controlled for other known or suspected risk factors for breast cancer in our multivariate models, thus decreasing the influence of confounding although our adjusted and unadjusted results did not differ substantially.

The main limitation of our study was the use of self-reported breastfeeding which may have led to measurement error; however, an earlier validation study by Kark *et al. *reported high concordance between breastfeeding derived from clinic records compared with an interview conducted 20 to 22 years later (Spearman *P *= 0.86) [29]. A role of recall bias is unlikely given that there is no reason for women to suspect a role of breastfeeding in the etiology of their disease. In addition, we were not able to differentiate between women who exclusively breastfed versus those who also supplemented with bottle feeding. Despite this, with 1,665 matched pairs, this is the largest study to date addressing the role of breastfeeding in the etiology of *BRCA*-associated breast cancer development.

## Conclusions

In summary, these findings corroborate a protective role of breastfeeding on breast cancer risk for *BRCA1*. The lack of an association for *BRCA2 *mutation carriers suggests that the biological pathway for carcinogenesis is different for the two genes. Women with a *BRCA *mutation should be advised of the benefit of breastfeeding in terms of reducing breast cancer risk.

## Abbreviations

BMI: body mass index; *BRCA1*: breast cancer susceptibility gene 1; *BRCA2*: breast cancer susceptibility gene 2; CI: confidence interval; HRT: hormone replacement therapy; OR: odds ratios.

## Competing interests

The authors declare that they have no competing interests.

## Authors' contributions

JK participated in study design, data analysis and interpretation, manuscript drafting and revision. JL, HTL, CKS, WDF, PG, SLN, RD, NT, PA, LS, AE, CE, CS, OG, JB, TH and AP participated in data acquisition and manuscript revision. LS participated in data interpretation, and in manuscript drafting and revision. PS participated in statistical analysis and interpretation of the data, while SAN conceived the study, participated in study design, data acquisition, data analysis and interpretation of the data, and in manuscript drafting and revision. All authors read and approved of the final manuscript.
